# Training neurologists for Brazil: from geographic imbalance to national responsibility

**DOI:** 10.1055/s-0046-1827823

**Published:** 2026-09-25

**Authors:** Eduardo Boiteux Uchôa Cavalcanti, Eduardo Genaro Mutarelli

**Affiliations:** 1 Rede SARAH de Hospitais de Reabilitação, Departamento de Neurologia, Brasília DF, Brazil.; 2 Universidade de São Paulo, Departamento de Neurologia, São Paulo, SP, Brazil.

Nascimento's article, A map of neurology medical residency in Brazil in 2025: really a nation?, raises a question that extends well beyond the distribution of training vacancies: whether the geographic distribution of specialist training is adequate for a country of continental dimensions. 1

In 2025, Nascimento documented 332 first-year positions across 101 neurology residency programmes; 51.8% were in the Southeast, with 30.7% in the state of São Paulo alone, whereas only four (1.2%) were in the North. Five states–Acre, Amapá, Rondônia, Roraima, and Tocantins–had no program at all. 1 Based on Nascimento's counts and 2024 regional population estimates reported in *Demografia Médica no Brasil* 2025, the South and the Southeast offered ∼2.2 and 1.9 first-year positions per million inhabitants, respectively—roughly 11 and 10 times the density recorded in the North (0.2 per million). 1 2

The distribution also mirrors Brazil's economic geography. In 2023, the Southeast and the South together generated 69.8% of national gross domestic product (GDP), and in 2025 they held 72.6% of neurology positions; the North and Northeast generated 19.6% of GDP and held 20.8% of positions. 1 3 The asymmetry is not merely administrative. Neurology reproduces the geography of the broader medical training system and, in the North, deepens it. 2 4

The unequal geography of neurology residency reveals how Brazil trains, distributes, and institutionalises neurological expertise. Residency does more than produce specialists: it shapes clinical reasoning, professional identity, research culture, and ethical responsibility. Where physicians train also predicts, in large measure, where they eventually practise. 5 Regions without neurology residency programmes therefore forfeit more than a training site. They lose the means to strengthen services, develop supervisors, generate local data, and form clinical and academic leaders.

Internationally, neurology residency is affected by variation in program duration, access to training resources, skill acquisition, particularly in low- and middle-income settings. Brazil shares these challenges, but with a distinctly geographic dimension. Santos- Santos-Lobato et al. showed that the central problem in the country is not an absolute shortage of neurologists but an unequal distribution that correlates with socioeconomic inequality. 4 *Demografia Médica no Brasil 2025* further demonstrates the value of demographic and residency indicators for workforce planning. 2 Nascimento's article extends this concern to the foundations of specialty training. 1

Expansion cannot, therefore, be a purely numerical exercise. Opening positions without adequate educational infrastructure risks converting an equity-driven policy into a formally accredited but educationally fragile one. A high-quality neurology residency requires a sufficient case mix, structured supervision, protected teaching time, and preservation of the clinical foundations of the specialty. As Nitrini has emphasized, technological access cannot substitute for clinical maturity, careful neurological assessment, or the doctor–patient relationship. 6

Training must also enable neurologists to appraise international evidence critically, recognize regional patterns of disease, and generate Brazilian epidemiological and health services research. Emerging programmes should therefore be integrated into national teaching networks and supported in producing local evidence to inform regional care, referral pathways, workforce planning and retention strategies. Evidence of low physician retention rates reinforces the importance of treating retention as a measurable outcome. 7 Without financial incentives, adequate infrastructure, and viable career pathways in underserved areas, expansion and retention will remain policy aspirations rather than functional realities.

Curricular quality and research capacity are inseparable from equity: a program that is geographically present but educationally deficient fails the communities it is meant to serve. Brazilian surveys have identified important gaps in electroencephalography education and in training for breaking bad news — core dimensions of diagnostic competence, communication, prognostication, and trust. 8 9 Competency-based medical education offers a framework that makes progression, supervision, and assessment explicit. 10 In Brazil, however, competency frameworks must be implemented as developmental commitments rather than as documentary requirements. Closing the quality gap across all programmes demands the same national commitment as closing the geographic one.

What should follow from this map? Brazil needs an updated public observatory linking neurology residency positions to demographic, epidemiological, socioeconomic, service-capacity, and retention indicators. These data should inform accreditation, financing, and regional service planning. Expansion should prioritise underserved regions, but only where appropriate educational conditions can be guaranteed. Established centres could support emerging programmes through interinstitutional rotations, visiting supervision, remote education, shared case conferences, and national teaching modules. Faculty development and local research capacity must become formal policy objectives.

Evaluation must likewise extend beyond vacancy counts to encompass occupancy, quality of supervision, resident progression, research participation, graduate practice location, retention, and contribution to regional care. Funding should reward sustained educational capacity and retention rather than program creation alone. This will require coordinated governance among regulatory, educational, and professional bodies, universities, hospitals, health managers, and residents.

A national neurology workforce cannot be built only where academic medicine is already strong; it must also be developed where neurological need is greatest. Brazil needs a residency system capable of correcting historical inequalities while preserving educational quality and strengthening local capacity. The map is before us. Whether it remains a portrait of imbalance or becomes the foundation of a deliberate national project is a responsibility shared by Brazilian neurology and the institutions that shape its future.
